# Novel CD123-aptamer-originated targeted drug trains for selectively delivering cytotoxic agent to tumor cells in acute myeloid leukemia theranostics

**DOI:** 10.1080/10717544.2017.1367976

**Published:** 2017-08-28

**Authors:** Haibin Wu, Meng Wang, Bo Dai, Yanmin Zhang, Ying Yang, Qiao Li, Mingyue Duan, Xi Zhang, Xiaomei Wang, Anmao Li, Liyu Zhang

**Affiliations:** a Shaanxi Institute of Pediatric Diseases, Xi’an Children’s Hospital, Xi’an, Shaanxi, People’s Republic of China;; b Key laboratory of Environment and Genes Related to Diseases, Ministry of Education, Xi’an Jiaotong University Health Science Center, Xi’an, Shaanxi, People’s Republic of China;; c Department of Orthopedics, The No.11 Hospital of PLA, YiNing, XinJiang, People’s Republic of China;; d Shaanxi Center for Stem Cell Application Engineering Research, Xi’an, Shaanxi, People’s Republic of China;; e Shaanxi Key Laboratory of Ischemic Cardiovascular Disease, Institute of Basic and Translational Medicine, Xi’an Medical University, Xi’an, Shaanxi, People’s Republic of China;; f Clinical Laboratory, Xi’an Children’s Hospital, Xi’an, Shaanxi, People’s Republic of China

**Keywords:** CD123 aptamer, targeted drug train, self-assembly, *in vivo*, high drug loading

## Abstract

Since conventional chemotherapy for acute myeloid leukemia (AML) has its limitations, a theranostic platform with targeted and efficient drug transport is in demand. In this study, we developed the first CD123 (AML tumor marker) aptamers and designed a novel CD123-aptamer-mediated targeted drug train (TDT) with effective, economical, biocompatible and high drug-loading capacity. These two CD123 aptamers (termed as ZW25 and CY30, respectively) can bind to a CD123 peptide epitope and CD123 + AML cells with high specificities and KD of 29.41 nM and 15.38 nM, respectively, while has minimal cross reactivities to albumin, IgG and trypsin. Further, TDT is self-assembled from two short primers by ligand-modified ZW25 that acted as initiation position for elongation, while intercalated by doxorubicin (Dox). TDT is capable of transporting high capacity of Dox to CD123 + cells and retains the efficacy of Dox, while significantly reducing drug uptake and eased toxicity to CD123− cells *in vitro* (*p* < .01). Moreover, TDT can ease Dox cytoxicity to normal tissues, prolong survivals and inhibit tumor growth of mouse xenograft tumor model *in vivo*. These suggest that CD123 aptamer and CD123 aptamer-mediated targeted drug delivery system may have potential applications for selective delivery cytotoxic agents to CD123-expressing tumors in AML theranostics.

## Introduction

Acute myeloid leukemia (AML) is a genetically and biologically heterogeneous disease (Ehninger et al., 2014; Medinger et al., 2016). The growth of uncontrolled abnormal clonal proliferation of myeloid precursors results in the accumulation of leukemic blasts, causing severe impairment of normal hematopoiesis (Jung et al., 2015; Mardiros et al., 2015). The clinical outcomes of AML have improved only minimally over the past three decades though (Yeung & Radich, 2017). Standard chemotherapy is still an essential option for the treatment of AML (Bose & Grant, 2015). However, a major problem associated with chemotherapy is relapse (Short & Ravandi, 2016), resulting in an overall 5-year-survival rate of only 30–40% (Du et al., 2007). The chemotherapeutic agents are unable to distinguish tumors from normal tissues. These features may generate serious problematic side effects such as limited drug intensity, duration of chemotherapy and reduced therapeutic efficacy (Cornelissen & Blaise, 2016; Yeung & Radich, 2017). These disadvantages may result in treatment failure, relapse and poor prognosis (Shlush & Mitchell, 2015; Benito et al., 2016; Tauro, 2016). To circumvent these problems, there is an urgent need to develop novel AML therapeutic strategies with higher efficacy and accuracy.

One approach is AML-targeted therapy, in which anticancer drugs can be selectively delivered to leukemia cells but not to their normal counterparts. In addition, the cytotoxicity to tumor cells is enhanced, while the efficacy of treatment is improved. Therefore, targeted therapy holds great promise in improving anticancer efficacy and a variety of targeting agents have been developed to achieve AML-targeted therapy (Khan et al., 2017). At present, antibodies displayed an attractive opportunity and some have entered phase-I clinical trials (Moradi-Kalbolandi et al., 2016; Liu et al., 2017; Wang et al., 2017; Xie et al., 2017). These data have showed that antibody talacotuzumab (CSL362) has great targeting ability, excellent tolerance and favorable safety, indicating antibodies as promising agents for AML-targeted therapy (Xie et al., 2017). However, antibodies have disadvantages that need to improve. For instance, some patients may become resistant to agents after preliminary treatment (Schoumacher & Burbridge, 2017), some patients’ immune system may be activated by antibodies and generate adverse effect. Most importantly, since sensitive to temperature, pH and multigelation, antibodies are easier to lose their functions, and these drugs are complicatedly designed, laboriously prepared, limited drug loaded and highly cost (Niraula & Ocana, 2016). Therefore, novel AML-targeted drug delivery system has advantages over existing method such as antibodies in treatment of AML is urgently needed.

A classic targeted drug delivery system usually consists of anticancer drugs and targeting ligands, which can specifically bind to tumor markers that overexpressed on the surface of cancer cells but with relatively low expression even no expression on normal cells and tissues (Zhang et al., 2016). The α subunit of IL-3 R (IL-3Rα, CD123), is a well-characterized 75 kD plasma membrane glycoprotein (Liu et al., 2015). CD123 is highly expressed on 45–95% of AML cells (Du et al., 2007), while at low or negative level on primitive hematopoietic stem cells, erythroid progenitor cells, mature granulocytes and lymphocytes (Liu et al., 2015). AML patients with higher CD123 were usually exhibited increased resistance to chemotherapeutic drugs (Ehninger et al., 2014) and have lower complete remission (CR) rate, higher relapse rate and negative prognosis (Thokala et al., 2016). Thus, CD123 is an ideal tumor marker for AML-targeted drug delivery system (Mardiros et al., 2015; Li et al., 2016; Ding et al., 2017). In addition, besides tumor marker, tumor-targeting ligand is also an important element for targeted cancer therapy. Despite antibodies, aptamers (apts) are new type of targeting ligands that show tremendous potential for clinical application (Mirian et al., 2017; Poolsup & Kim, 2017). Aptamers are short single-strand oligonucleotides, which can bind to target molecules with high affinity and specificity (Prodeus et al., 2015). Aptamers are generated by systematic evolution of ligands by exponential enrichment technique (SELEX) (Sett et al., 2017). SELEX was invented by Craig Tuerk and Larry Gold in 1990 (Tuerk & Gold, 1990), which is one of the most effective methods to generate aptamers for targets (Robertson & Joyce, 1990; Bock et al., 1992). Compared with antibodies, aptamers possess distinctive advantages over targeting ligands: high affinity for binding to target molecules (Lyu et al., 2016), limited synthesis cost, small sizes that allows them to penetrate solid tumors (Soldevilla et al., 2015) and nonimmunogenic (Chen et al., 2017), which may facilitate long-term therapeutic efficacy and safety. Most noteworthy is that on account of geometrical conformational flexibility and synthesis dynamics, aptamers can be readily synthesized and chemically modified for various therapeutic applications. In consequence, aptamers have been employed as novel targeting ligands in drug delivery systems against prostate cancer (Jing et al., 2016; Hao et al., 2016; Atabi et al., 2017), breast cancer (Taghavi et al., 2017), pancreatic cancer (Yoon et al., 2017), lung cancer (Holmboe et al., 2017; Li et al., 2017) and leukemia (Bahreyni et al., 2017; Jang et al., 2017). Aptamers have emerging and exhibited amazing tumor-targeting abilities for targeted drug delivery systems (Zhu et al., 2013a).

Anticancer agents are the major components of targeted drug delivery system for AML treatment. Generally, aptamer-mediated targeted drug delivery systems are aptamer–drug conjugates or aptamer–nanomaterial complexes (Li et al., 2011; Herrmann et al., 2014; Mardiros et al., 2015; Xie et al., 2017). These modalities are mostly aptamers directly linked with molecular anticancer agents or nanoparticles parceling anticancer drugs (Petros & DeSimone, 2010; Mosafer et al., 2017; Pang et al., 2017). However, these systems own special limitations, which become the biggest obstacle that inhibit their application translations to clinic. Firstly, these complex are programed complicatedly, the preparation of sophisticated nucleic acid-based nanomaterials or aptamer–drug conjugates are laborious. This problem may hamper the production scale-up (Chang et al., 2011; Zhu et al., 2013b). Secondly, the drug-loading capacities are limited (Douglas et al., 2012), resulting in insufficient drug uptake for tumor cells. This disadvantage is unable to kill tumor cells completely and tumor cell subclones resistant to drugs will survive, which will cause replase (Jiang et al., 2012). Thirdly, the materials used in nanoparticels are usually poorly biodegradable, causing chronic accumulation in tissues. Lastly, since nanoparticles are much larger than aptamers, the three dimensional structures of aptamers may be influenced seriously, even lose their specific binding abilities. Thus, to circumvent these limitations, we designed an aptamer-mediated targeted drug train (TDT). This aptamer-originated DNA TDT is self-assembled by two probes rich in C/G base and a pair of trigger probes linked to aptamer, through a hybridization chain reaction. The ‘train’ will be long enough after hybridization. Since Dox can intercalate in C/G base pair, they may load onto TDT with high concentration and efficiency. Thus, TDT owns the ability of loading Dox furthest (Wei et al., 2017). We hypothesise that TDT can bind to targeted cells selectively due to aptamer; afterwards, it will be internalized and degraded. Finally, Dox is released into nuclei and can interfere DNA replication and inhibit tumor growth *in vivo*.

So far, there is no literature reported on CD123 aptamers, or CD123 aptamer-mediated TDT platform. In this study, we try our first attempt to develop CD123 aptamer by SELEX technique, we have successfully selected two 66-based DNA aptamer (termed ZW25 and CY30) against an extracellular peptide epitope of CD123. Specifically, we tested the binding specificities of ZW25 to CD123+ and CD123− cells. Further, CD123 aptamer ZW25-mediated TDT was constructed. It is found that TDT could selectively bind to CD123 + cells and enhance growth inhibition to CD123 + tumor cells prominently both *in vitro* and *in vivo*. Meanwhile, TDT reduced drugs adverse effects and prolonged survivals of animal models *in vivo*. Overall, these results prove that CD123 aptamer ZW25 is a potential targeting molecule in AML-targeted therapy, and CD123 aptamers-mediated targeted drug delivery systems are great options for AML-targeted therapy.

## Materials and methods

### Reagents

ssDNA library for SELEX and primers were synthesized by Sangon Biotech (Shanghai China). CD123 peptides with at least 95% purity were synthesized by SBS Genetech (Beijing China). Bovine serum albumin (BSA, Sigma-Aldrich, Japan, Catalogue#:V900933), trypsin (Sigma-Aldrich, Japan, Catalogue#: T2600000), IgG (Sigma-Aldrich, US, Catalogue#: I4506), cell counting kit 8 (CCK8, Sigma-Aldrich, US, Catalogue#:96992), streptavidin-coated magnetic beads (Promega, Madison, WI), carboxyl-modified magnetic microspheres (Beaver, Suzhou, China Catalogue#: 70102-50), Transferrin-Alexa633 (Thermal Fisher Scientific, US, Catalogue#:T23362), 1-ethyl-3-(3-dimethyllaminopropyl)-carbodiimide hydrochloride (EDC, Sangon Biotech, Shanghai, China, Catalogue#:C600433). N-Hydroxysuccinimide (NHS, Sangon Biotech, Shanghai, China, Catologue#:C100219), doxorubicin (Dox, Sangon Biotech, Shanghai, China, Catologue#: A603456), TA cloning kit (TransGen Biotech, Beijing, China, Catalogue#:CT101).

### Cell lines and cell culture

Human acute myelocytic leukemia (Molm-13) and human B-cell precursor leukemia (RCH-ACV) were gifted from Xi’an JiaoTong University (China). Human erythroleukemic cell line (TF1, Cat# CRL-2003) and mouse lymphoma cell line (EL4, Cat#TIB-39) were obtained from ATCC (US). Cells were cultured in RIMP-1640 medium supplemented with 20% fetal bovine serum (FBS) and a mixture of penicillin/streptomycin. Cells were grown at 37 °C in humidified atmosphere with 5% of CO_2_. All experiments were performed on cells in exponential growth phase.

### Fixation of target on magnetic beads

The target of SELEX is a 24-AA peptide with the sequence of TDIECVKDADYSMPAVNNSYCQFG. This sequence is a part of CD123 extracellular domain predicted from Protein Data Bank (PDB). We modified a disulfide bond between two cysteines which are underlined to simulate its natural structure maximally. The conjugation of the target to magnetic beads was accomplished via cross linking of -COOH and -NH_2_. To fix SELEX target, 6 × 10^5^ carboxylated magnetic beads were washed by 200 μL MES (100 mM, pH 5.0) at room temperature twice. Then, these magnetic beads were activated by 100 μL 1-ethyl-3-(3-dimethyllaminopropyl)-carbodiimide hydrochloride (EDC) (20 mg/mL) and 100 μL N-hydroxysuccinimide (NHS) (20 mg/mL) for 15 min with gentle stirring. Next, beads were washed by linking buffer (5.3 mL 0.2 M sodium dihydrogen phosphate and 94.7 mL 0.2 M sodium hydrogen phosphate). 5 μg CD123 peptides was added to the beads and incubated at room temperature for 2 h. At last, the magnetic beads were washed three times with PBS buffer and stored at 4 °C. Similar method was employed to conjugate the beads with other substances, including BSA, IgG and trypsin.

### SELEX library and primers

A random DNA library was constructed of 66-mer oligonucleotides with 28-base long randomized in central and fixed sequences at both sides. The sequence of library was 5′-TGCGTGTGTAGTGTGTCTG-(N28)-CTCTTAGGGATTTGGGCGG-3′, in which *N* represents a randomized nucleotide of either A, T, C or G. To obtain FITC/FAM-labeled sense single-strand DNA, FITC/FAM-labeled forward primer (5′-TGCGTGTGTAGTGTGTCTG-3’) and biotin-labeled reverse primer (5′- CCGCCCAAATCCCTAAGAG-3′) were used in PCR for the synthesis of double-labeled DNA (dsDNA) molecules. Then, the dsDNA was mixed with streptavidin-coated magnetic beads for 20 min at room temperature with gentle agitation. Magnetic beads were washed with PBS buffer twice and denatured in alkaline condition (0.1 M NaOH). The FITC-labeled ssDNA was separated from the biotin-labeled antisense ssDNA strand by magnetic used for aptamer selection or detection.

### SELEX procedure *in vitro*

To furthest improve selection efficacy and reduce selection nonspecificity, we added a step before typical SELEX. The procedures of SELEX were described below. Firstly, before selection rounds, the ssDNA (50 pM) was heated up to 95 °C for 5 min and cooled on ice immediately. These ssDNA were incubated with BSA (1 mg) for 30 min to maximally exclude nonspecific sequences. Secondly, ssDNA were incubated with CD123 peptide-coated beads at 37 °C with gentle stirring for 30 min. To reduce background, 0.1 mg/mL salmon sperm DNA and 1 mg/mL of BSA were added to binding buffer. Thirdly, after incubation, the unbound oligonucleotides were removed by washing four times with 300 mL PBS buffer. Subsequently, bead–ssDNA complex were amplified by PCR with FITC- or biotin-labeled primers (25 cycles of 30 s at 95 °C, 30 s at 56 °C, 30 s at 72 °C, followed by 10 min at 72 °C). Lastly, dsDNA from PCR was separated into ssDNA via procedure described earlier. The FITC-ssDNA was used for the next round of SELEX.

### Flow cytometry analysis

To monitor the selection efficiency of SELEX and enrichment of aptamers, the FITC-labeled ssDNA pool was incubated with CD123 peptide-coated magnetic beads in 200 μL PBS buffer at 37 °C for 30 min after each selection round. The beads were washed twice with 0.2 mL of binding buffer and then resuspended in 0.1 mL of PBS buffer. The FITC fluorescence was determined with a FACS caliber cytometer (BD). BSA, IgG and trypsin-coated beads were assessed as negative protein. After several rounds of selection, when the ssDNA-targeting CD123 were fully enriched, the selected ssDNA pool was PCR-amplified using unmodified primers and cloned into *Escherichia coli* with TA cloning kit (Cat# CT101) for DNA sequencing.

### Aptamer-binding characteristics assays

To assess the binding specificity of aptamers to target, the FITC-labeled aptamer was separately incubated with CD123 peptide-, BSA-, IgG- or trypsin-coated magnetic beads. The beads were washed twice, resuspended in 0.1 mL PBS buffer and analyzed by flow cytometry.

The binding affinity of aptamers was evaluated by incubating CD123 peptide-coated magnetic beads with gradient concentrations of FITC-labeled aptamer at 37 °C for 30 min. The beads were washed three times and resuspended in 0.2 mL PBS buffer and subjected to flow cytometric analysis. The BSA-coated magnetic beads were used as negative controls to measure nonspecific binding. All of the experiments for binding assay were repeated three times. The mean fluorescence intensity of target labeled by aptamers was used to calculate for specific binding by subtracting the mean fluorescence intensity of nonspecific binding to BSA-coated beads. The equilibrium dissociation constants (*K*_d_) of the aptamer-CD123 interaction were obtained by fitting the dependence of fluorescence intensity of specific binding on the concentration of the aptamers to the equation: (Davis et al., 1996).

               Y=B max X/(Kd+X)

To evaluate whether aptamer could bind to CD123+ cells selectively, The FAM-labeled aptamer (60 pM) was separately incubated with either 1 × 10^5^ Molm-13, TF-1, RCH-ACV or EL4 cells at 37 °C for 30 min. Then, the cells were analyzed by flow cytometry (BD FACS Calibur, NJ).

### Construction of TDT

Aptamer ZW25 was linked to a ligand to trigger multiple annealing (Table S1). Probes P1(4 nM), P2(4 nM) and ZW25-ligand (10 nM) were mixed and annealed programed (95 °C 2 min, and decreasing 0.1 °C per 8 s, until down to 25 °C), then left at room temperature overnight. Thus, ZW25-OCDLS have been prepared, and the products were assessed by 1.0% agarose gel. Further, to construct drug-loaded TDT, ZW25-OCDLS was incubated in an aqueous solution of Dox (5 nM) for 30 min in a black 96-well plate at a range of aptamer/dox molar ratios. The drug loading was monitored by fluorescence spectrometry by Synergy4 analyzer (UK) (lEx =488 nm, lEm =500–700 nm) (Liu et al., 2012).

### Binding assay of ZW25-OCDLS by confocal microscopy imaging and flow cytometery

The specific binding of TDT to CD123 + cells was studied by flow cytometry and confocal fluorescence scanning microscopy. CD123 + Molm-13 cells (1 × 10^6^ in 200 μL of medium) were determined by incubating with 2 mM FAM-labeled ZW25-OCDLS at 37 °C for 4 h (Zhou et al., 2016). CD123- RCH-ACV cells were used as control. The resultant cells were washed and resuspended in 100 μL mounting medium. All cell fluorescent images were scanned with a Leica TCS SP5 confocal microscope (Leica Microsystems).

For flow cytometry analysis, Molm-13 cells were collected from the culture bottles and washed twice with PBS buffer. 1 × 10^6^ Molm-13 cells were incubated with either 200 nM FAM-labeled ZW25-OCDLS, FAM-labeled random library, FAM-labeled ZW25 or FAM-labeled P1 mixed with P2 for 1 h at 37 °C, respectively, and washed twice with PBS buffer. RCH-ACV cells were used as CD123− control. All cells were analyzed with flow cytometry.

### Stability evaluation of TDT by dialysis assay

TDT (50 μM Dox equivalent, 300 μL) and Free Dox (50 μM, 300 μL) were transferred into dialysis device [3.5 molecular weight cutoff (MWKO); Float-A-Lyzer® G2 G235065]. Each device was infused in 5 mL of PBS buffer in an individual dialysis peeper, with special stirring (150 rpm/min) at the bottom of each device. At the indicated time points, a 100 μL sample was collected from each device for Dox fluorescence measurement [Ex: 480 nm; (Em): 590 nm] using Synergy4 analyzer (UK).

### TDT internalization assay using confocal microscopy imaging

The internalization of TDT to CD123 + cells were studied by confocal fluorescence scanning microscopy. Molm-13 cells (2 × 10^5^) in PBS buffer (200 μL) were incubated with TDT (2 mM) at 37 °C for 2 h. The internalization was terminated by putting cells on ice. RCH-ACV cells were used as CD123− control cells. Cells were washed and stained with Alexa 633 for 15 min. All cell fluorescent images were scanned on a Leica TCS SP5 confocal microscope (Leica Microsystems, Buffalo Grove).

### *In vitro* cytotoxicity assay

*In vitro* cytotoxicity was determined using Cell Counting kit8 (Sigma-Aldrich, US, Cat#96992) to evaluate the cytotoxicity of TDT or free Dox against CD123 + and CD123− tumor cells. Firstly, to explore the most effective ZW25-OCDLS/Dox ratio at which TDT could the most effectively targeted damage CD123+ tumor cells, both CD123 + cell lines Molm-13 and TF-1(5 × 10^4^ cells per well) were first seeded in 96-well plates and treated with TDT at a range of ZW25-OCDLS/Dox molar ratios (1:50, 1:20, 1:10, 1:8, 1:5, 1:3 and 1:1) for 1 h at 37 °C. Then, cells were washed with PBS buffer and cultured for a further 48 h. Afterwards, CCK8 assay was used to determine cell viability per standard protocol outlined by the manufacture’s instruction. At which ratio that cells had the lowest viabilities was the most effective ratio, TDT with this ratio should be applied for following experiments. Secondly, to assess the targeted damage ability to CD123 + tumor cells of TDT, CD123 + cell lines Molm-13 and TF-1, CD123- cell lines EL4 and RCH-ACV (5 × 10^4^ cells per well) were all first seeded in 96-well plates and treated either with TDT or free Dox at the concentration of 5 mM at 37 °C for 1 h. Then, cells were washed with PBS buffer and cultured for a further 48 h. Afterwards, CCK8 assay was used to determine the cell viability per standard protocol outlined by the manufacture’s instruction.

### Evaluation of TDT-reduced cytotoxicity to normal tissues *in vivo*

C57 mice were purchased from the Fourth Military Medical University Lab Animal Centre. To investigate TDT’s cytotoxicity to normal tissues, mice were treated with either TDT, free Dox as positive control, or saline as negative control by s.c., with six mice in each group. The body weight of each mouse was measured each day to monitor the drug toxicity. The blood of each groups was collected and assessed for serum marker of organ damage (CK-MB, AST, ALT and BUN) and serum-associated inflammatory cytokines (TNF-α and INF-α). All mice were euthanized at day 25. The heart, liver, kidney, spleen, stomach and lung tissues were collected and employed for hematoxylin and eosin (H&E) staining to evaluate TDT’s adverse effects to normal tissues.

### Evaluation of TDT anticancer effects *in vivo*

BALB/c mice were purchased from the Fourth Military Medical University Lab Animal Centre and raised under pathogen-free conditions. To evaluate the anticancer ability of TDT, the mouse xenograft tumor model was developed by s.c. injecting 2 × 10^7^ *in vitro*-propagated Molm-13 cells (in 100 μL of PBS buffer) into BALB/c mice. Dorsal tumor nodules were allowed to grow to a volume of ∼100 mm^3^ before treatment initiation. Tumor-bearing mice were randomly divided into three groups, with six in each group: (i) treated with TDT; (ii) treated with free Dox; and (iii) treated with saline. The Dox dosage was kept in group ii at 2 mg/kg, and the TDT dosage in group i was accordingly maintained the same to that in group ii. Drugs were supplemented through tail vein injection every day. Tumor volume was calculated using the following equation:

$$\text{Tumor volume}=\text{Tumor Length}\times {\text{Tumor Width}}^{\text{2}}/\text{2}.$$

### Statistics

All numerical data were expressed as the mean ± S.D. Statistical differences between two groups were determined by the Student’s *t*-test. *p* < .05 was considered statistically significant.

## Results

### Hypothesis of TDT for selectively delivering dox to AML tumor cells

**Figure 1.**
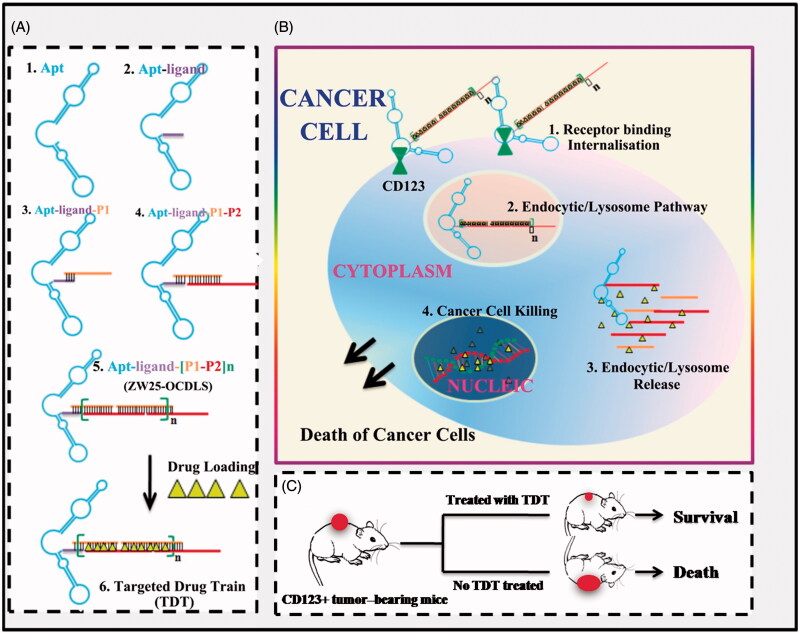
Schematics of self-construction of aptamer-orientated DNA TDT for targeting transportation of chemotherapeutics in theranostic applications. (A) Self-construction of TDT from short DNA aptamer ZW25: (1) The initial ZW25. (2) The ZW25 chimeric that linked to a trigger ligand. (3) Initiation from the first hybridization probe P1. (4) The further construction with hybridization probe P2. (5) The resultant long strands. (6) Drug loading onto TDT. (B) The drugs were specifically transported to target cancer cells via receptor binding between CD123 and aptamer ZW25: (1) TDT was targeted binding to CD123+ cancer cells and internalized via the binding specificity of aptamer ZW25 to CD123 protein. (2) TDT was transferred by endocytic/lysosome vacuole. (3) endocytic/lysosome vacuole was burst and TDT was degraded by enzyme. (4) Dox was released within nuclei and induced cell death. (C) TDT could inhibit tumor growth and prolong survivals.

This CD123-aptamer-originated TDT platform is a self-assembled system by two probes rich in C/G base and a pair of trigger probes linked to aptamer, through a hybridization chain reaction. Afterwards, TDT will be long enough after hybridization and owns plenty of ‘boxcars’. Since Dox can intercalate in C/G base pair, they may load onto these ‘boxcars’ of TDT with high concentration and efficiency (Figure 1(A)). Thus, TDT owns the ability of loading Dox furthest. Further, we hypothesis that TDT can targeting bind to CD123 + AML cells selectively due to CD123 aptamer. Afterwards, TDT will be internalized by CD123 + cells via endosome/lysosome pathway. TDT is degraded and Dox is released into nuclei and can interfere DNA replication (Figure 1(B)). As a consequence, TDT can inhibit tumor growth *in vivo* (Figure 1(C)).

### Aptamer selection process and monitoring

**Figure 2.**
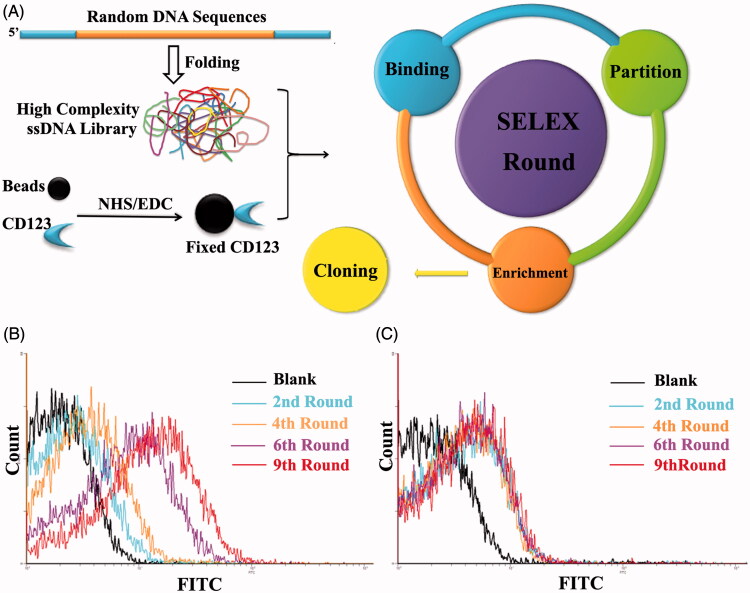
Schematic representation of SELEX system. A. Random DNA sequences contains 66-mer oligonucleotides with 28-base long randomized in central and fixed sequences at both sides. When folded into a three-dimensional structure, it became a high complexity ssDNA library that could satisfy SELEX completely. CD123 peptides were fixed on magnetic beads by NHS/EDC reaction. SELEX round contains binding, partition and enrichment. After several rounds, the pool was cloned. B. Flow cytometry monitoring of the enrichment of aptamers. Compared with the starting random DNA pool, flow cytometry revealed an increase in fluorescence intensity of aptamers bound to the CD123 peptide after the second, the fourth, the sixth and ninth rounds of selection. C. Flow cytometry monitoring of the nonspecific binding to BSA of aptamers.

The target of SELEX process was a 24-AA peptide of CD123 protein that located at the extracellular domain. It is reported that this sequence is exposed on cells as the most immune-dominant peptide epitope (Broughton et al., 2014a, 2014b). To simulate its natural structure maximally, we linked a disulfide bond between the two cysteine amino acid residues. This CD123 peptide was covalently conjugated to magnetic beads by EDC/NHS reaction. The random ssDNA pool employed contained 66-mer oligonucleotides. When folding into three-dimensional structure, this high complexity ssDNA library could generate at least 10^15^ species of sequences. This diversity could fulfill SELEX procedure completely (Figure 2(A)). To monitor the efficacy of SELEX, the enrichment of aptamers was assessed by flow cytometry and agarose gel electrophoresis. When compared with random ssDNA from the initial pool, there is an increasing amount of ssDNA bound to target-coated magnetic beads after each round of selection (Figure 2(B,C)). The ssDNA targeting to CD123 were furthest enriched at the 9th selection round and the DNA pool was subsequently cloned. 60 clones were performed for further function evaluations.

### Identification and characteristic of CD123 aptamers

**Figure 3.**
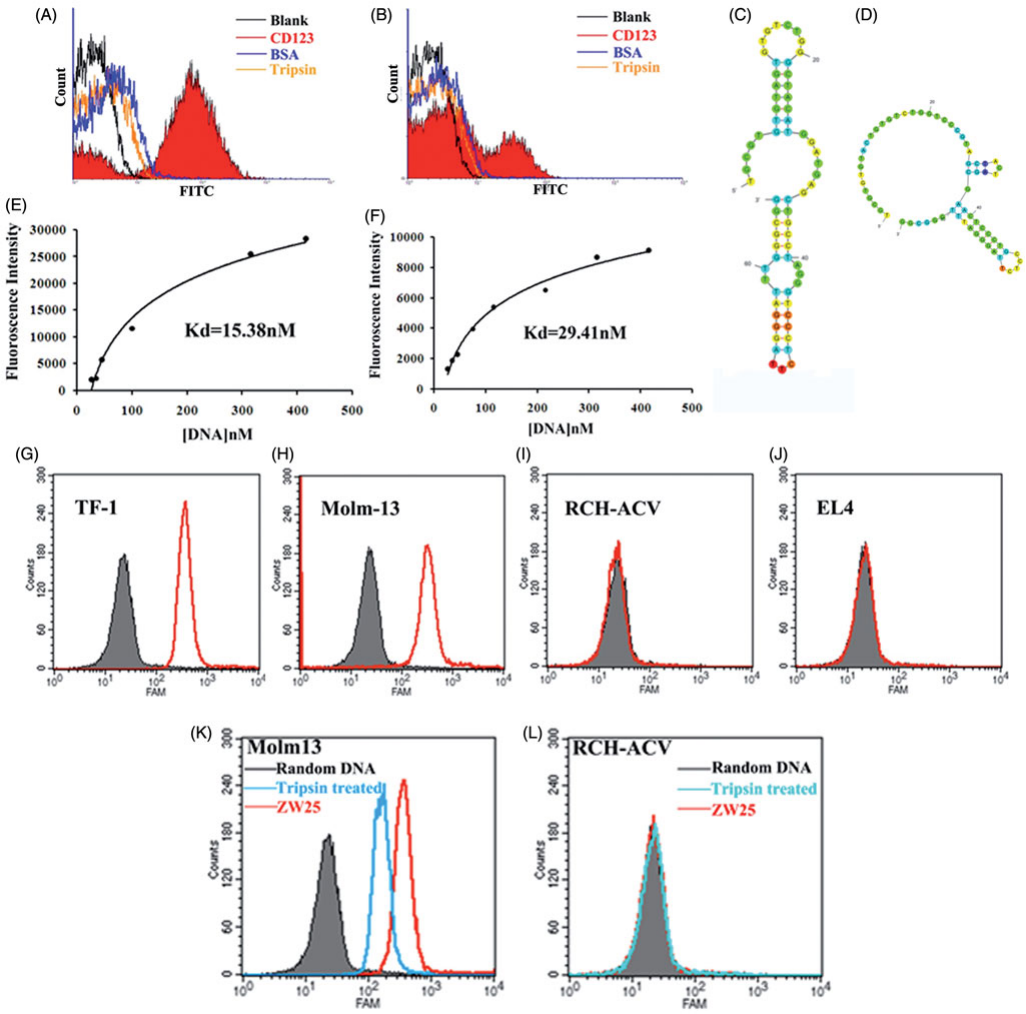
Characterization of aptamers ZW25 and CY30. (A,B). Flow cytometry evaluation of the binding specificity to CD123-peptide or other negative proteins by FITC-labeled aptamers ZW25 and CY30. FITC-labeled aptamers ZW25 (A) or CY30 (B) was incubated with CD123 peptide beads, BSA beads and trypsin beads separately. The black curves represent the control fluorescent signals generated by FITC-labeled random DNA from the library pool. (C,D). The predicted secondary structure of ZW25 and CY30. The secondary structures of aptamer ZW25 (C) and CY30 (D) was predicted by M-fold software. (E,F). Flow cytometry evaluation of the binding affinities to CD123-peptide by FITC-labeled aptamers ZW25 (E) and CY30 (F). (G–J). Flow cytometry to assess ZW25’s binding specificity to CD123 + and CD123− cells. The histograms were generated after incubating FAM-labeled ZW25 with TF-1 (G), Molm-13 (H), RCH-ACV (I) and EL4 (J) cells, respectively. The filled histograms represent the control fluorescence signals generated by FAM-labeled random DNA from the library pool. (K,L). Flow cytometry evaluation of ZW25’s binding affinity to CD123 + and CD123− cells treated by trypsin. CD123 + cell line Molm-13 (K) and CD123− cell line RCH-ACV (L) were treated with trypsin or PBS for 5 min and treated with FAM-labeled aptamer ZW25. Control fluorescent signal (gray filled) was generated by untreated Molm-13 or RCH-ACV cells incubated with FAM-labeled random DNA.

Among 60 clones, two aptamers termed ZW25 and CY30 exhibited relatively high binding affinity to the target CD123 peptide. To evaluate whether ZW25 or CY30 could bind to CD123 peptides selectively, binding specificity tests were performed. Since BSA, IgG and trypsin are abundant in blood, we examined aptamer ZW25 and CY30 binding to these three proteins. Specifically, BSA, IgG, trypsin or CD123 peptides coated beads were incubated with FITC-labeled ZW25 or CY30, and analyzed by flow cytometry. As shown in Figure 3, both ZW25 (Figure 3(A)) and CY30 (Figure 3(B)) showed a significant binding to CD123 peptides, but a relatively weak binding to BSA, IgG or trypsin. The data indicated that, ZW25 and CY30 had a lower cross reactivity to BSA, IgG, or trypsin compared to CD123 peptide.

To assess these two aptamers’ structure and thermodynamic parameters, their secondary structures were predicted by M-fold website (Figure 3(A,B)). The thermodynamic parameters of ZW25 and CY30 showed that they were quite stable (Supplementary Table S1). To quantitatively evaluate the binding affinity of ZW25 and CY30 to CD123 peptides, beads coated with CD123 peptides were incubated with either FITC-labeled ZW25 or CY30 at various concentrations. Using nonlinear regression analysis, ZW25 was found to have a KD of 29.41 nM and CY30 was 15.38 nM (Figure 3(E,F)). Aptamer ZW25 was applied for further investigations. Further, to assess whether aptamer ZW25 could recognize CD123 + tumor cells, FAM-labeled ZW25 was incubated with either CD123 + (Molm-13 and TF-1) or CD123 − (EL-4 and RCH-ACV) cell lines. Each cell line incubated with FAM-labeled random DNA was treated as control. The cells were then analyzed by flow cytometry. The fluorescence intensities were collected and presented in Figure 3(G–J). For CD123+ cell lines Molm-13 and TF-1, it is obvious that ZW25 treatment generated a significant fluorescence signals compared with random DNA treatment (Figure 3(G,H)). On the contrary, for CD123− cell lines EL-4 and RCH-ACV, fluorescence signal in ZW25 treated and random DNA treated are almost the same (Figure 3(I,J)). Therefore, it is indicated that aptamer ZW25 could bind to CD123 + tumor cells superiorly, and ZW25 could recognize CD123 structure.

To further investigate the influence of protein expression to ZW25 binding, CD123 + cell lines Molm-13 and TF-1 were predigested by trypsin for 5 min at 37 °C. Then, cells were incubated with FAM-labeled ZW25 and analyzed by flow cytometry. The fluorescence intensity in digested Molm-13 was significantly decreased when compared with non-digested cells (Figure 3(K,L)). The fluorescence intensity in either digested RCH-ACV (Figure 3(J)) or undigested was the same. It is suggested that the CD123 expression on CD123 + cells could influence the binding ability of ZW25 to CD123 + cells.

### Construction and characterization of TDT

**Figure 4.**
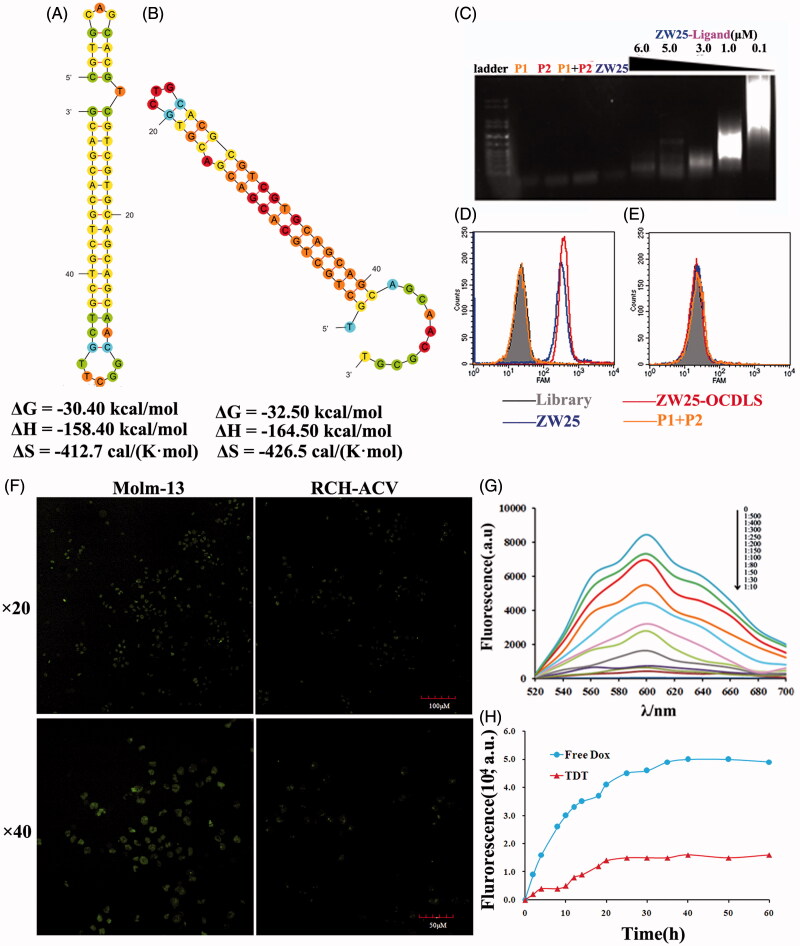
Characterization of the construction, selective cancer cell binding of TDT. (A,B). Analyzing secondary structure and thermodynamics of the P1 (A) and P2 (B) by M-fold software. C. Agarose gel electrophoresis indicating the self-assembly of ZW25-OCDLS derived from ZW25-ligand. (D,E). Flow cytometry results showing the selective recognition abilities of ZW25-OCDLS to Molm-13 cells (D), but not to RCH-ACV cells (E). F. Confocal microscopy images displaying the selective binding of ZW25-OCDLS to Molm-13 and RCH-ACV cells. Cells were incubated with ZW25-OCDLS (15,000 nM ZW25-ligand equivalents) at 37 °C for 4 h (P1 and P2: labeled with FAM). (Scale bars: ×20:100 μm; ×40: 50 μm.) G. Fluorescence spectra of doxorubicin solution (5 μM) with increasing molar ratios of the ZW25-OCDLS (from top to bottom: 0, 1:500, 1:400, 1:300, 1:250, 1:200, 1:150, 1:100, 1:80, 1:50, 1:30 and 1:10). The fluorescence quenching indicates Dox loading into ZW25-OCDLS. H. Dotted data points showing the fluorescence intensities of Dox diffused from free Dox or TDT (Dox: 50 μM) during dialysis to outside PBS buffer at different time points.

**Figure 5.**
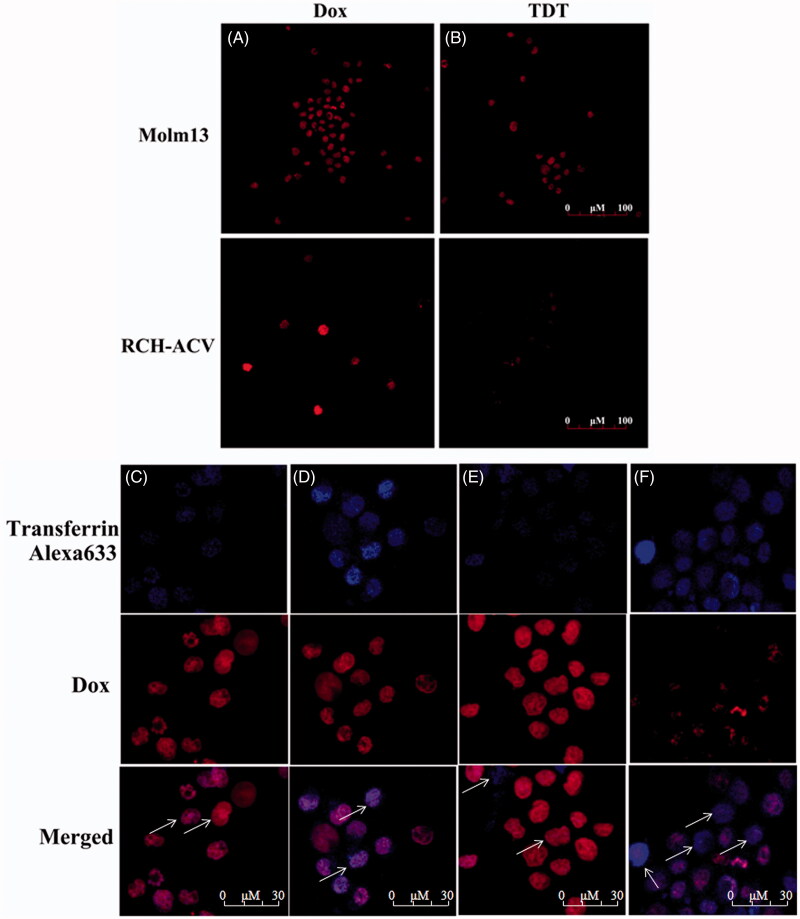
Dox’s selective delivery by TDT to CD123 + tumor cells (Molm-13) and monitoring of intracellular drug unloading via endocytosis/lysosome pathway. (A,B) Confocal laser scanning microscopy images of Molm-13 (A) and RCH-ACV cells (B) after incubation with free doxorubicin or TDT for 4 hours. (C–F) Confocal laser scanning microscopy images of TDT-mediated selective drug delivery to CD123 + tumor cells via endocytosis/lysosme. CD123 + Molm-13 cells (C and D) and CD123− RCH-ACV cells (E and F) were treated either with free Dox (C and E; 2 mM) or TDT (D and F; 2 mM Dox equivalents) for 2 h, followed by transferrin-Alexa 633 staining (arrows indicated probative cells; Scale bar: 30 μm).

According to the hypothesis, to construct the TDT platform, two elongation probes (P1, P2, sequences in Supplementary Table S2) were designed and synthesized. These two probes could fold into two hairpin structures stably and self-stored enough energy. Thus, they were protected by the corresponding stems and could prevent self-polymerization in the absence of an initiation probe (Figure 5(A,B)). To construct TDT, the ‘train’ for loading drugs should be constructed firstly. Aptamer ZW25 was modified on its 5′-end to trigger the self-assembly. Introduction of ZW25-ligand to probe P1 and P2 could trigger the hybridization, causing the self-assemble ZW25-oriented DNA strands (Figure 1(A)). To evaluate whether construction succeeded, 1% agarose gel electrophoresis was employed. Probe P1(4 μM), or P2(4 μM) or mixture of P1(4 μM) and P2(4 μM) or mixture of ZW25(1 μM) with P1(4 μM) and P2(4 μM), will not cause any hybridization (Figure 4(C)). In the presence of ZW25-ligand with various concentrations (0.1 μM, 1.0 μM, 3.0 μM, 5.0 μM and 6.0 μM,), the elongation was processed successfully. The molar ratio of ZW25-ligand to hybridization probes in the initial reaction mixture was investigated, and with increased ZW25-ligand concentration, the hybridization products became shorter.

To evaluate whether this ZW25-OCDLS could still selectively recognize CD123 + tumor cells, FAM-labeled probe P1, P2, ZW25, ZW25-ligand and ZW25-OCDLS were incubated either with CD123 + Molm-13 or CD123− RCH-ACV tumor cells. FAM-labeled P1, or P2 was treated as negative control and ZW25 was treated as positive control. ZW25-OCDLS and ZW25 could generate a significantly strong fluorescence signal on Molm-13 cells (Figure 4(D)), whereas a relatively weak signal on RCH-ACV cells (Figure 4(E)), and P1 with P2 group generated weak signal on both Molm-13 and RCH-ACV cells. Further, FAM-labeled ZW25-OCDLS incubated with CD123 + Molm-13 or CD123− RCH-ACV tumor cells were scanned by confocal microscopy. Molm-13 cells presented a relatively strong fluorescence on cell membranes whereas RCH-ACV did not (Figure 4(F)). It was indicated that ZW25-OCDLS remain the binding specificity to CD123 structure and could recognize CD123 + cells indeed. Therefore, ‘train’ was constructed successfully. Next, as Dox was widely used in clinical treatment, we employed Dox as a model drug in our further study. TDT was constructed by intercalating Dox into the ZW25-OCDLS. It is well-known that free Dox could emit fluorescence and be quenched after intercalating into DNA. Therefore, to assess whether Dox was incorporated into ZW25-OCDLS effectively, specifically, various concentrations of ZW25-OCDLS were mixed with Dox (5 μM) and the fluorescent signals were assessed by fluorescence spectroscopy. As shown in Figure 4(G), sequential decreases in the native fluorescence spectrum of Dox were observed when the concentration of ZW25-OCDLS was increased. When the DNA/Dox molar ratio is below 1:50, the fluorescence spectrum of Dox was at the lowest level and did not change further, indicating that most dox had incorporated into DNA structure at this ratio.

The stability of TDT is an essential parameter for clinical application. Next, to assess the stability of TDT, a drug diffusion experiment was processed [3.5 molecular weight cut off (MWKO); Float-A-Lyzer® G2]. Both TDT and free Dox were placed in dialysis bag respectively with gentle stirring (150 rpm/min) at the bottom of each device. At the indicated time points, a 100 μL sample was collected from each device for Dox fluorescence measurement. Free Dox solution diffused at a rapid speed in early 15 h whereas Dox in TDT diffused in much more slow speed, indicating high stability of TDT (Figure 4(H)).

### TDT for selective transporting of Dox into CD123 + tumor cells by endosome/lysosome pathway

Free Dox may be uptaken by both tumor and normal cells, which is a primary cause for its adverse effects against normal tissues. We assumed that when Dox is intercalated into the DNA structure of TDT, it is much more difficult to diffuse and can be preferentially delivered to CD123+ cancer cells. To test this hypothesis, the selectivity of TDT for targeted transport Dox to CD123 + tumor cells was evaluated by drug uptake assay in Molm-13 and RCH-ACV cells (Figure 5). Cells were treated with TDT or free Dox, respectively, followed by confocal microscopy examination. Molm-13 or RCH-ACV cells were treated with either free Dox, as a control, or TDT (ZW25-OCDLS/Dox molar ratio =1:40). Strong fluorescence signals were both observed in Molm-13 and RCH-ACV cells treated with free Dox (Figure 5(A,B)). In addition, strong fluorescence signals were shown in Molm-13 treated with TDT or free Dox, and RCH-ACV treated with free Dox. However, the fluorescence signal in RCH-ACV treated with TDT was much weaker. Therefore, it is suggested that TDT could selectively deliver Dox to CD123+ but not CD123− cells, whereas free Dox had no specificity to cells. Further, since we doubted that this selective delivery was internalized by endocytosis/lysosme pathway, transferring-Alexa 633 was used to locate the endosomes. As shown in Figure 5(C–F), the colocation of Dox and transferring-Alexa 633 was observed in Molm-13 (Figure 5(D)) but not RCH-ACV cells (Figure 5(F)) treated with TDT or Molm-13 cell (Figure 5(C)) and RCH-ACV cells (Figure 5(E)) treated with free Dox, indicating that TDT might be internalized by endocytosis/lysosme pathway. Taken together, TDT could deliver Dox selectively to CD123-posotive tumor cells, providing the basis for targeted therapeutics.

### TDT inhibit CD123+ tumor selectively and reduced cytotoxicity to CD123− cancer cells both *in vitro* and *in vivo*

According to the results above, TDT could deliver Dox selectively to CD123 + cells, we thus speculated that the cytotoxicity to CD123− cells would be decreased dramatically. To test this hypothesis, firstly, we should explore the best ZW25-OCDLS/Dox molar ratio of TDT that could the most effectively inhibit CD123+ tumor cells. TDT at a range of molar ratio were incubated with Molm-13 and TF-1 cells, and CCK8 kit was applied to assess cell viabilities. As shown in Supplementary Figure 1, when the ZW25-OCDLS/Dox molar ratio of TDT was 1:10, both CD123+ tumor cell lines were inhibited maximally. Thus, TDT that with ratio 1:10 (ZW25-OCDLS/Dox) was applied for further study.

**Figure 6.**
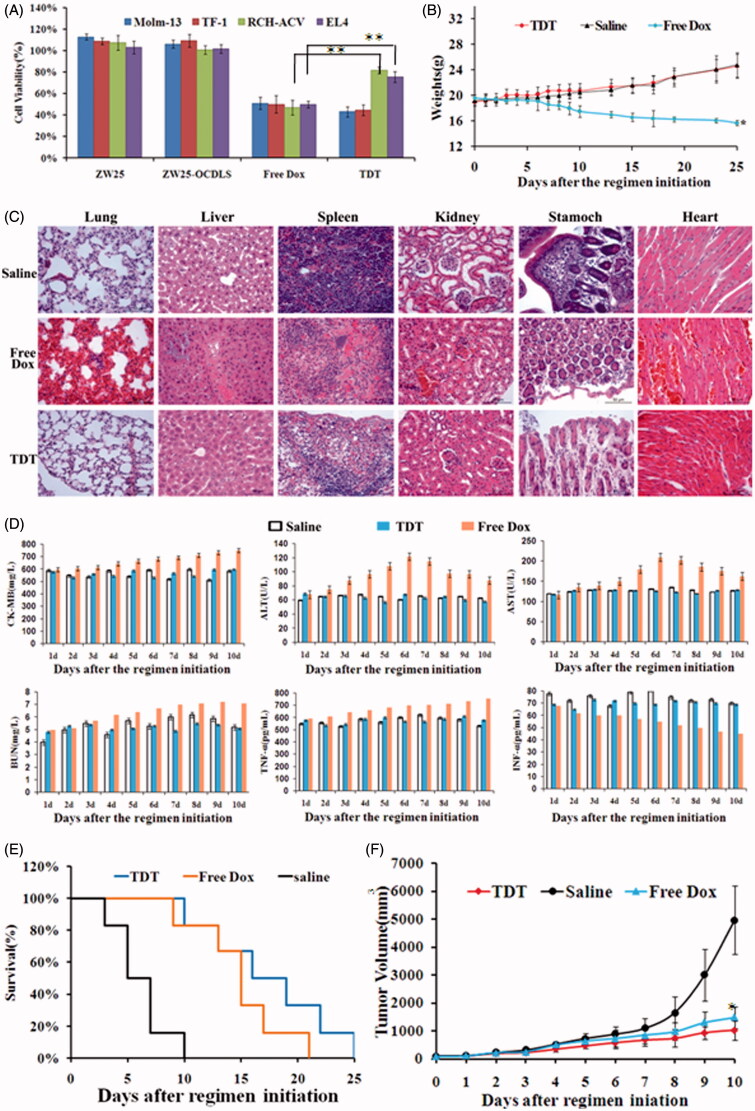
Potential reduced side effects and anticancer ability of TDT *in vitro* and *in vivo*. A. Cell viability assays *in vitro* after the cells were treated with CD123 aptamer ZW25, ZW25-OCDLS, free Dox or TDT for 3 h. The cell viability of Molm-13, TF-1, RCH-ACV and EL4 cells were evaluated by CCK8 assay after 48 h of further incubation (mean ± SD, *n* = 6, *p < .05). (B,C). TDT reduced cytotoxicity to normal tissues *in vivo*. Mice were randomly divided into two groups and treated either with TDT or saline. B. Weights up to 25 days after treatment initiation (mean ± SD; *n* = 6). C. 25 days after initial treatment, mice were euthanized and the heart, liver, kidney, spleen, stomach and lung tissues were observed by H&E staining. D. Serology assessment. A. Serum markers of organ damage. Each bar represents means with SD of six replicates. (E,F) TDT-targeted ability to inhibition CD123 + tumor growth. Molm-13 xenograft mouse tumor model was developed by s.c. injection of Molm-13 cells in the back of Babl/c mice. Mice were divided into three groups randomly, in which the following regimens with different agents were administered by i.v. injections each day: (i) TDT, (ii) free Dox (2 mg/kg), and (iii) saline (*n* = 6). E. Survival rate of mice after treatment initiation. F. Tumor volumes of mice after treatment initiation.

Secondly, to investigate whether TDT could targeted delivery anticancer drugs to CD123 + tumor cells and avoid damaging CD123− cells, CD123+ cell line Molm-13 and TF-1, CD123− cell line RCH-ACV and EL4 were treated either with TDT, ZW25-OCDLS, ZW25 or free Dox. CCK8 kit was subjected to cell viability test. For RCH-ACV and EL4 cells, the cell viabilities in TDT group were much stronger than free Dox group (*p* < .01), whereas there was no difference between TDT and free Dox in Molm-13 and TF-1cells (Figure 6(A)). Four cell lines treated with ZW25-OCDLS showed no dramatic decrease of cell viability. These results suggested that TDT tends to reduce the damage to CD123− cells while retaining the efficacy of Dox against CD123+ cells. It is worth noticing that ZW25-OCDLS alone was nontoxic towards both cell lines, and thus, it is relatively safe to cells and the cytotoxic was principally caused by Dox.

To further study whether TDT could reduce cytotoxity to normal tissues *in vivo*, the weights of each mouse after agents administration were measured. The mice ponderal growth in TDT was almost the same compared to the saline control group, however, the ponderal growth in free Dox group was inhibited (Figure 6(B)). After euthanization, the heart, liver, kidney, spleen, stomach and lung tissues of the mice were collected and stained by H&E. There was no obvious damage detected in the TDT group when compared with the saline group (Figure 6(C)), whereas free Dox group revealed an obvious damage of tissues. The serum markers of organs and serum-associated inflammatory cytokines assessed in TDT group were similar when compared with the saline group (Figure 6(D)). However, the serum markers of organs and serum-associated inflammatory cytokines assessed in free Dox group were higher (*p* < .05). We thus concluded that TDT could reduce cytotoxity to normal cells and tissues.

To investigate the targeted anticancer efficacy of TDT *in vivo*, we developed animal xenograft model by s.c. injecting Molm-13 cells into BALB/c mice. When tumors grew to ∼100 mm^3^ in average, mice were divided into three groups randomly, in which the following regimens with different agents were administrated by i.v. injections each day: (a) TDT, (b) free Dox (2 mg/kg), and (c) saline. Dox dosage used in tests was determined according to the results reported. TDT and free Dox resulted in a longer survival time than saline, the elongation of survival time indicated the potential anticancer effects of TDT (Figure 6(E)). Further, tumor volumes were measured each day. Tumor volumes in TDT and free Dox group were significantly decreased when compared with the saline group (Figure 6(F)), and TDT group showed smaller tumor sizes and longer survival. Detailed study of this would be our interest in the future. Overall, these data demonstrated the potent antitumor efficacy via TDT.

## Discussion

Chemotherapy is still a major clinical approach to treat AML (Medinger et al., 2016), whereas its efficacy is largely limited since chemotherapeutics are unable to distinguish cancer cells from normal cells, resulting in damages to normal cells and tissues. One of the strategies to alleviate these adverse effects is tumor targeted therapy (Medinger et al., 2016), which could targeted delivers anticancer agents to tumor cells and avoid nonspecific damage to normal cells maximum. CD123 is considered as a valuable target for AML targeted therapy due to its over-expression in most AML cells. Aptamers can bind to target with high affinity and specificity and may be constructed as targeting ligand for novel selective drug delivery system. In this study, using SELEX technology and an extracellular peptide of CD123 protein as target, we first developed CD123 aptamers ZW25 and CY30, with KD of 29.41 nm and 15.38 nm respectively. It was found that these two aptamers could recognize CD123 peptide and CD123+ cells selectively. Further, we constructed the first, novel, effective CD123 aptamer-mediated targeted drug train (TDT), a self-assembled drug loading platform. This TDT could carry and targeted deliver abundant anticancer drugs Dox to CD123+ AML cells with high specificity and accuracy. There was the colocation of Dox and endosome in Molm-13 cells treated with TDT, whereas no such overlap in CD123 + cells treated with free Dox, or CD123- cells treat with either TDT or free Dox. It is indicating that the hydrophilic aptamer prevented TDT from freely defuzing into the lipid cell membrane, but via a receptor-mediated endocytosis. That is, ZW25 within TDT recognized CD123 structure on cell surface and bound to CD123 + cells, resulting in activating of endosome pathway. CCK8 assays indicated that TDT selectively delivered Dox to CD123+ AML cells and inhibited proliferation *in vitro*. Further, TDT alone have no toxicity to normal tissues and cells *in vivo* and significantly prolonged mean averaged survival time and decreased tumor volumes of CD123+ tumor-bearing mice compared with mice treated with saline *in vivo*.

Generally, the most effective method to develop aptamers is SELEX (Tuerk & Gold, 1990). The targets applied in SELEX are usually peptides for synthetic peptides are much purer than proteins. However, the choice of peptides is of great importance. If the three-dimensional structures of peptides are not the same as natural proteins in unison, or if the locations of peptides are buried inside the proteins, thus, aptamers selected for peptides may not recognize proteins, which will hamper the application in clinic. In order to develop aptamers recognize CD123 protein, a 24-AA peptides (TDIECVKDADYSMPAVNNSYCQFG) within the extracellular domain as our target for SELEX. This part has been identified as the most immune-dominant peptide epitope within extracellular domain (Broughton et al., 2014a, 2014b). To maximally simulate the natural structure of this peptide, we linked two cysteines with disulfide bond. Further, peptides as SELEX targets are superior to proteins or cells because it could be synthesized with high purity, providing the most effective SELEX process. After nine rounds of selection, we have successfully selected two aptamers termed ZW25 and CY30, which could recognize CD123 peptide selectively. It is interesting that ZW25 could bind to CD123 + AML cells preferentially as well. We speculated that this might be related to the following reasons: Firstly, this peptide is a predicted epitope that is in the extracellular domain and external part of CD123 protein. The crystal structure of CD123 reveals that the extracellular domain of this protein is always in an extended conformation. Secondly, it is confirmed that this peptide is within the immune-domain that binds to CD123 antibody CSL362 (Broughton et al., 2014 b). Since this peptide is exposed on the surface of CD123 protein, aptamers that are able to bind to this peptide with high specificity and affinity may be capable of binding to CD123+ cells as well.

Nowadays, AML-targeted therapies are limited. There have been no drugs approved in over four decades. The potential application of novel AML-targeted strategies in clinical treatment is one of the most essential parameters indeed. Aptamers, used as novel specific recognition ligands, have been studied for targeted conventional anticancer agent delivery systems for cancer-targeted therapy (Poolsup & Kim, 2017). Plenty of researches have proved that aptamer-mediated targeted drug delivery systems can facilitate targeted delivery of anticancer agents to tumor cell both *in vitro* and *in vivo*. Here, in this study, we have designed the first CD123 aptamer-mediated TDT. Importantly, the CD123 aptamer-mediated periodically hybridization DNA strands provide a large number of spatially addressable sites, allowing high-capacity loading of therapeutics or agents. Moreover, this TDT is programable, periodic, biodegradable and self-assembled. These features are expected to reduce the cost for DNA preparation, limit maximum tolerated dose MTD, reduce side effects, improve therapeutic efficacy in cancer therapy, providing unprecedented opportunities for clinical applications (Zhu et al., 2013 b). In our study, TDT could targeted deliver anticancer drugs Dox into CD123+ tumor cells. When compared with free Dox, the anticancer efficacy of TDT is more effective and targeting ability of TDT made it much safer to normal tissues and cells. The nontargeted damage from TDT to normal tissues is more alleviative than free Dox. It should be noted that the more Dox TDT carried, the more effective anticancer ability of TDT theoretically. As our data showed, when this ratio is 1:50, the fluorescence of Dox was at the lowest level and did not change further, indicating that most Dox had incorporated into DNA structure at this ratio. ZW25-OCDLS loaded the most Dox as it could, which means TDT owns the most powerful ability to damage CD123+ tumor cells. However, our data showed that when ZW25-OCDLS/Dox molar ratio is 1:10, the anticancer abilities are greatest than other ratio. We speculated that there is a balance between ZW25-OCDLS and Dox. When Dox occupied ZW25-OCDLS fully, the targeting ability of TDT will be influenced and reduced. Dox loaded on TDT may leak quicker, and these leaked Dox will damage normal cells, and Dox delivered to CD123+ cells may be insufficient. When ZW25-OCDLS is far more than Dox, although the leak of Dox may be much slower and the damage to normal cells will be alleviative, the amount of Dox on TDT is far from adequate to targeted and inhibit CD123+ tumor cells. Thus, we made the ZW25-OCDLS/Dox molar ratio of TDT as 1:10 in the further study.

Nevertheless, extensive future research is still needed to improve the nuclease resistance of TDT, drug-loading capacity and targeted ability. Meanwhile, future animal tests should be focused on the pharmacokinetics of TDT and to answer other preclinical questions.

## Conclusions

In summary, the de novel two CD123 aptamers ZW25 and CY30 were found capable of binding to CD123 peptide and CD123 + AML tumor cells, with minimal binding to CD123− cells. Further, to assess whether ZW25 could applied as targeting ligand to selectively transport anticancer agents to CD123 + cells, we constructed a ZW25-medited TDT system, which was easily designed and prepared and with high drug payload capacity. This TDT could targeted transport Dox to CD123 + cells via endosome pathway and reduced the nontarget cytotoxicity to CD123− cells while remained the inhibition to CD123+ cells *in vitro*. Animal tests have shown that TDT could significantly prolong the survival elongation and inhibit tumor growth of CD123+ tumor-bearing mice and alleviate side effects to normal tissues *in vivo*. Collectively, these features are poised to make CD123 aptamer ZW25 uniquely attractive for AML-targeted drug delivery system, and it is obvious that CD123 aptamer-mediated TDT has great potential application for the development of novel AML-targeted drug delivery system.

## Supplementary Material

IDRD_Zhang_et_al_Supplemental_Content.pdf
