# Supplementary material for: Novel CD123-aptamer-originated targeted drug trains for selectively delivering cytotoxic agent to tumor cells in acute myeloid leukemia theranostics
Source: Drug Deliv. 2017 Aug 28;24(1):1216–29. doi: 10.1080/10717544.2017.1367976 (PMC8241133; doi:10.1080/10717544.2017.1367976)
Supplement: IDRD_Zhang_et_al_Supplemental_Content.pdf [file IDRD_A_1367976_SM0721.pdf]

Table S1 Thermodynamic analysis of CD123 aptamers. Thermodynamic analysis of ZW25 and CY30 were evaluated by M-fold website, including free Gibbs energy, enthalpy change, and entropy change.

**Seq.ID**	**Sequence**	**Free Gibbs Energy(ΔG)（kcal/mol at 37℃）**	**Enthalpy change (ΔH) (kcal/mol)**	**Entropy change (ΔS)(cal/k.mol)**
**ZW25**	TGCGTGTGTAGTGTGTCTGGGC<br>TACATCGATGAGCTGCCTAGGG<br>TCCCTCTTAGGGATTTGGGCGG	**-20.20**	**-106.10**	**-276.9**
**CY30**	TGCGTGTGTACTGTGTCTGGTC<br>CCGTAGCTACTAGCGAACTCCC<br>TGCCTCTTAGGGATTTGGGCGG	**-10.60**	**-62.20**	**-166.3**

Table S2 Sequences of hybridization probes. FITC, or FAM, if necessary, were modified at the 5'-ends of P1 and P2.

Probes	Sequences(5'→3')
1. Ligand	TGCTGCTGCACGACGACGTGCTGCACGTTT
2. P1	CGTGCAGCACGTCGTCGTGCAGCAGCAACGGCTTGCTGCTGCTGCACGACG
3. P2	TGCTGCTGCACGACGACGTGCTGCACGCGTCGTGCAGCAGCAGCAACGCGT

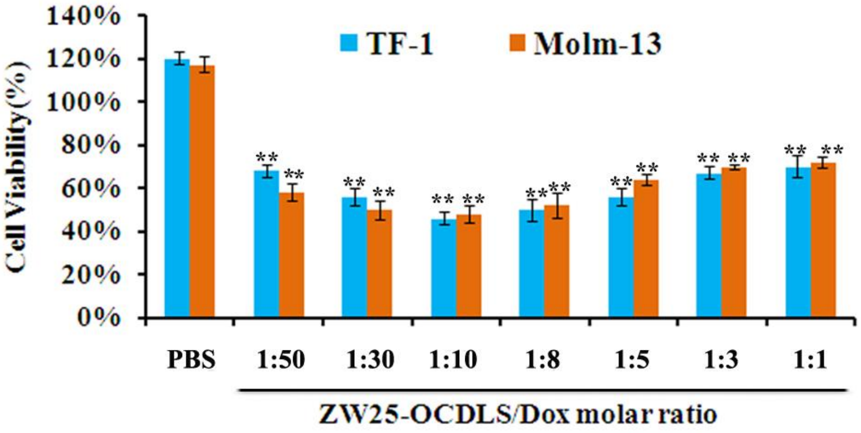

Supplementary Figure 1. The assessment of TDT's anticancer ability with various ZW25-OCDLS/Dox molar ratios. CD123+ cell lines Molm-13 and TF-1 were treated with various ZW25-OCDLS/Dox molar ratios (1:50, 1:30, 1:10, 1:8, 1:5, 1:3 and 1:1). CCK8 kit was applied to evaluate cell viabilities. Cells treated with PBS were treated as control. (**indicates P<0.01).
